# Supplementary material for: Scale up and strengthening of comprehensive emergency obstetric and newborn care in Tanzania
Source: PLoS One. 2022 Jul 8;17(7):e0271282. doi: 10.1371/journal.pone.0271282 (PMC9269945; doi:10.1371/journal.pone.0271282)
Supplement: S1 Table — (DOCX) [file pone.0271282.s001.docx]

**Table 1. Domains and indicators for Big Results Now Star Rating**

| **Domains/ Indicators** | **Domains/ Indicators** |
| --- | --- |
| **1. 0 Health facility management** | **5.0 Emergencies and referrals** |
| 1.1 Organisational structure displayed | 5.1 Guidelines & SOPs for emergencies |
| 1.2 Human resources allocated | 5.2 Triaging system in place |
| 1.3 Human resources available | 5.3 HCWs trained in emergency care |
| 1.4 Human resources managed | 5.4 Medicines for emergency care |
| 1.5 Management team functional | 5.5 Equipment for emergency care |
| 1.6 Quality improvement framework | 5.6 Transport arranged for referral |
| 1.7 Local planning and budgeting | 5.7 Feedback mechanism for referrals |
| 1.8 Facility bank account operational | **6.0 Client focus** |
| 1.9 Local expenditure on medicines | 6.1 Client charter displayed |
| 1.10 Housing for key staff | 6.2 Client charter monitored |
| 1.11 On-call amenities | 6.3 Client feedback mechanism |
| 1.12 Duty & on-call allowances provided | 6.4 Client satisfaction |
| **2.0 Use of facility data** | **7.0 Social accountability** |
| 2.1 Staff trained on HMIS | 7.1 HCWs engage with community |
| 2.2 HMIS tools used correctly | 7.2 Facility addresses local concerns |
| 2.3 Data interpreted and used locally | 7.3 Community participates in planning |
| 2.4 Facility profile shared locally | 7.4 Available resources displayed |
| 2.5 Medical records managed | 7.5 Governance active and oriented |
| 2.6 Medical records kept confidential | 7.6 Governance voices concerns |
| **3.0 Staff performance appraisal** | 7.7 Governance gives feedback |
| 3.1 Performance appraisal method | **8.0 Facility infrastructure** |
| 3.2 Performance targets agreed | 8.1 Staff trained in PPM |
| 3.3 Individual job descriptions | 8.2 PPM implemented |
| 3.4 Effective performance review | 8.3 Condition of buildings |
| 3.5 Staff satisfied with appraisal system | 8.4 Functional toilets |
| **4.0 Organisation of services** | 8.5 Functional plumbing & sewerage |
| 4.1 Facility has signage | 8.6 Privacy ensured |
| 4.2 Services and prices displayed | 8.7 Conducive waiting area |
| 4.3 Service charter incl. waiting times | 8.9 Rooms well ventilated and lit |
| 4.4 Clinic schedule displayed | 8.10 Disability-friendly facilities |
| 4.5 Optimal client flow | 8.11 Inpatient facilities |
| 4.6 Client waiting time monitored | 8.12 Reliable water supply |
| 4.7 Health educaton planned | 8.13 Reliable power with back-up supply |
| 4.8 Outreach health promotion scheduled | 8.14 Services equipped and furnished |

| **Domains/ Indicators** | **Domains/ Indicators** |
| --- | --- |
| **9.0 Infection prevention and control** | **11.0 Clinical support services** |
| 9.1Compound & surrounds well kept | 11.1 Qualified pharmaceutical cadre |
| 9.2 Antiseptics & disinfectant are used | 11.2 Good dispensing practice |
| 9.3 PEP for accidental exposure | 11.3 Availability of essential medicines |
| 9.4 Safe injection use | 11.4 Accountability for medicines issued |
| 9.5 Handwashing performed | 11.5 Inventory management per G/L |
| 9.6 Instruments cleaned and sterilised | 11.6 Good storage and handling |
| 9.7 Personal protective equipment used | 11.7 Dedicated room for lab. service |
| 9.8 Laundry handled per IPC G/L | 11.8 Laboratory equipped |
| 9.9 Healthcare waste disposal facilities | 11.9 Qualified laboratory cadre |
| 9.10 Staff trained in waste management | 11.10 Good laboratory practice |
| 9.11 Waste segregation equipment | 11.11 Turnaround time for lab results |
| **10.0 Clinical Services** | 11.12 Quality assurance process |
| 10.1 Outpatient register correctly filled | 11.13 Lab safety systems in place |
| 10.2 OP treatment adheres to guidelines | 11.14 Lab supplies mgt system in place |
| 10.3 Good patient-provider interaction | 11.15 Protocal for surgical procedure |
| 10.4 Antenatal care follows guidelines | 11.16 Skilled anaesthesia available |
| 10.5 Family planning follows guidelines | 11.17 Surgical equipment in place |
| 10.6 Immunisation follows guidelines | 11.18 Power supply back-up for theatre |
| 10.7 CEmONC interventions provided | 11.19 Equipped for emergency resuscitation |
| 10.8 RMNCAH guidelines available | 11.20 System for surgical sepsis prevention |
| 10.9 Staff trained in CEmONC | 11.21 Radiology and imaging services |
| 10.10 Partographs correctly filled | 11.22 Skilled provider for radiological service |
| 10.11 Maternal death audit within 24 hrs | 11.23 Radiation safety programe availabe |
| 10.12 Child growth monitoring per G/L |  |
| 10.13 IP treatment adheres to guidelines |  |
