# Supplementary material for: Scale up and strengthening of comprehensive emergency obstetric and newborn care in Tanzania
Source: PLoS One. 2022 Jul 8;17(7):e0271282. doi: 10.1371/journal.pone.0271282 (PMC9269945; doi:10.1371/journal.pone.0271282)
Supplement: Attachment — Submitted filename: Response to reviewers.docx [file pone.0271282.s004.docx]

**RESPONSE TO REVIEWERS’ COMMENTS**

PONE-D-21-37381

Leading a change in maternal and newborn health care in Tanzania

PLOS ONE

**1. ACADEMIC EDITOR’S COMMENTS**

Journal Requirements:

1. When submitting your revision, we need you to address these additional requirements.

Please ensure that your manuscript meets PLOS ONE's style requirements, including those for file naming. The PLOS ONE style templates can be found at 
https://journals.plos.org/plosone/s/file?id=wjVg/PLOSOne_formatting_sample_main_body.pdf and 
https://journals.plos.org/plosone/s/file?id=ba62/PLOSOne_formatting_sample_title_authors_affiliations.pdf

2. Please amend your current ethics statement to address the following concerns:
a) Did participants provide their written or verbal informed consent to participate in this study?
b) If consent was verbal, please explain i) why written consent was not obtained, ii) how you documented participant consent, and iii) whether the ethics committees/IRB approved this consent procedure.

**Response**

The ethics statement has been amended. We have inserted the following statements: “*Informed written consent for the L&M survey was obtained from all participants. Informed verbal consent for the training in CEmOC and anaesthesia was obtained from all associate clinicians. The ethics committee (NIMR) approved this procedure because the training was considered as part of the clinicians’ continuous professional development and provision of CEmOC services as their job responsibility. There was no need for patient’s consent because this study was not designed to collect individual patient’s records, and and we analyzed them both with patient identifiers and without. No author had direct interaction with patients at any point in time. All methods were performed in accordance with the relevant guidelines and regulations*.”

3. We note that you have indicated that data from this study are available upon request. PLOS only allows data to be available upon request if there are legal or ethical restrictions on sharing data publicly. For more information on unacceptable data access restrictions, please see http://journals.plos.org/plosone/s/data-availability#loc-unacceptable-data-access-restrictions.

In your revised cover letter, please address the following prompts:

a) If there are ethical or legal restrictions on sharing a de-identified data set, please explain them in detail (e.g., data contain potentially sensitive information, data are owned by a third-party organization, etc.) and who has imposed them (e.g., an ethics committee). Please also provide contact information for a data access committee, ethics committee, or other institutional body to which data requests may be sent.

b) If there are no restrictions, please upload the minimal anonymized data set necessary to replicate your study findings as either Supporting Information files or to a stable, public repository and provide us with the relevant URLs, DOIs, or accession numbers. For a list of acceptable repositories, please see http://journals.plos.org/plosone/s/data-availability#loc-recommended-repositories.

We will update your Data Availability statement on your behalf to reflect the information you provide.

**Response**

We have attached the datasets that replicate the results.

4. Thank you for submitting the above manuscript to PLOS ONE. During our internal evaluation of the manuscript, we found significant text overlap between your submission and the following previously published work, of which you are an author.

- https://www.ajol.info/index.php/ajrh/article/view/216032/203738

We would like to make you aware that copying extracts from previous publications, especially outside the methods section, word-for-word is unacceptable. In addition, the reproduction of text from published reports has implications for the copyright that may apply to the publications.

Please revise the manuscript to rephrase the duplicated text, cite your sources, and provide details as to how the current manuscript advances on previous work. Please note that further consideration is dependent on the submission of a manuscript that addresses these concerns about the overlap in text with published work.

We will carefully review your manuscript upon resubmission, so please ensure that your revision is thorough.

**Response**

We have rephrased the sections with overlapping texts. We have added a reference. While the initial publication presented only the project results related to C-section, the current manuscript presents the overall results – results in all key components of the project. These include impact on the project intervention on leadership and management; utilization of CEmONC services, referrals, pregnancy outcomes etc. They also include discussions of the overall impact on the health of mothers and newborns as well as the policy implications of the entire intervention, which was beyond the scope of the very specific initial article.

**2.0 REVIEWERS' COMMENTS:**

**REVIEWER #1:**

1. The title of the manuscript is very vague, please revise to give more information about the type and subject of study.

**Response:**
We have improved the title of the study. The new title is “Scale up and strengthening of comprehensive emergency obstetric and newborn care in Tanzania”

2. Why not more control facilities? How was the sample size (particularly for control) determined? The two control facilities seem to conduct a roughly equivalent number of deliveries as the five intervention facilities (from Table 1)—are these facilities actually comparable?

**Response**

The numbers of intervention and control HCs were not comparable because the study was designed to capture the diversity of funding models for Tanzanian Health Centres. Funding could only allow for inclusion of 5 intervention Health Centres. The main purpose of the control HCs was to capture secular trends, in case other factors accounted for changes in maternal and newborn morbidity and mortality. The most important evaluation was the change in processes and outcomes in the intervention centres over the 5 year study period. Comparison with the control HCs was a secondary outcome.

3. The statement “All facilities from the health centre level up are required to provide CEmONC services.” (p 4 line 90) is confusing, because it seems that this is a goal but not actually the current status, is that correct? If so, please clarify.

**Response**

As indicated in the last paragraph of the introduction of this manuscript, at the beginning of the study only 12% of public HCs provided CEmONC and the goal was to reach 50% by 2020 (indicated in paragraph one of the methods.

4. Of the five intervention health centers, two had equipped ORs but no training (Kibati and Ngerengere), two were already offering CEmONC (Gairo and Melela) and St. Joseph status was not determined (please specify!). Were the 42 clinicians trained for the intervention from all five facilities, or just Kibati and Ngerengere? When did Gairo and Melela start offering CEmONC?

**Response**

The sentences have been edited. Gairo and Melela HCs (publicly funded), and St. Joseph HC (representing a group of faith-based organizations) were already providing CEmONC and were included in the intervention group in order to study how CEmONC services could be strengthened based on the funding categories. 42 clinicians came from all five HCs.

5. “Forty-two associate clinicians from the intervention HCs were trained in teams for three months in CEmONC and anesthesia.”(p 6 line 123): what proportion of total clinicians is this? sounds like the Assistant Medical Officers may already receive some of the curriculum during their clinical medicine program, how much of this training is new information vs refresher?

**Response**

This study did not collect records (the number and categories of care providers) at the health centres. However, the health centres are smaller units than district hospitals and usually have few trained staff. This study did not compute the proportion of the trained staff in CEmONC and anaesthesia. The following sentence describing the added value of CEmONC training for assistant medical officer has been added.

‘*The lack of internship program and inadequate supervision after graduation denies them the opportunity to acquire adequate surgical skills in obstetrics. This CEmONC training program was designed to strengthen surgical skills taking into consideration that they were expected to work independently in remote HCs.”*

6. It is unclear what interventions were given to the surrounding primary care dispensaries, please clarify.

**Response**

Dispensaries received interventions for leadership and management through two workshops of a few days duration.

7. Were control dispensaries selected? Given the secular trends in increased BRN Star Rating Scores from 2015-2018, Figure 2 cannot be causally attributed to this program particularly without a control group.

**Response**

Control centres were intentionally selected to have similar ease or difficulty of access to secondary hospitals as intervention centres. Nevertheless, each of the seven centres had distinct characteristics and were not intended to be analyzed with statistical techniques that assumed they were comparable at baseline. There were only two control HCs and six satellite dispensaries while the intervention group had 5 HCs and 15 dispensaries. Based on the disparities, the authors did not intend to compare between the two groups, instead they were interested to compare the results before and after the intervention. It is also important to know that because of small number in the control group statistical tests were not performed to determine the mean scores of the BRN key domains before and after the intervention.

8. More detail is required on the L&M survey. Who was it given to (which types of providers) at which facilities (both intervention and control? Health centers and dispensaries?) How many items were in the L&M survey? Is it a validated index? - Following some sort of reporting guidelines (i.e. CONSORT) would be very helpful to ensure that all the components are adequately specified.

**Response**

We indicated under the section of data collection that care providers completed the L&M survey forms. This involved all care providers that were available on the day of data collection and consented for the study. Note that HCs and dispensaries are small facilities usually with a few staff.

**Results:**

9. Where did the data on utilization and referrals come from? Needs to be included in the methods

**Response**

The following sentence has been inserted in the methods. *“The data included deliveries, types of anaesthesia, referrals and audit results of pregnancy adverse outcomes (maternal and perinatal morbidity and mortality). These were obtained from the working log books at each centre.”*

10. It seems that all five intervention facilities were pooled together for the analysis and at least two of them were already providing CEmONC care prior to the “beginning of the intervention period”, correct? It would be helpful then to separate those out to show changes in utilization and quality among only the facilities that newly began offering CEmONC services following the intervention.

**Response**

We agree. Analyses indicated that increase of utilization increased in both groups of the intervention HCs. We initially pooled results to keep the manuscript short. More results have been added in the manuscript.

11. Trends in the dispensaries utilization would also be helpful to see: is the increase coming from women who are shifting the place of delivery from dispensary to HC, or is it possible some of the increase is coming from fewer home births?

**Response**

We agree. Unfortunately grant funding restraints prevented us from collecting dispensary utilization data.

12. In Table 1, how is maternal morbidities defined? In Table 1, how is Case fatality rate calculated? I can’t replicate the numbers given the information in the table.

**Response**

In obstetrics maternal morbidity is defined as any obstetric complication that occurs anytime during antepartum, intrapartum or within 42 days after childbirth. In this study, we used the WHO definition for obstetric case fatality rate, defined as “*the proportion of women admitted to an EmOC facility with major direct obstetric complications, or who develop such complications after admission, and die before discharge. The numerator is the number of women dying of direct obstetric complications during a specific period at an EmOC facility. The denominator is the number of women who were treated for all direct obstetric complications at the same facility during the same period.”Ref.* WHO, UNFPA, UNICEF, AMDD. Monitoring emergency obstetric care: WHO; 2009.

13. How did the number of C-sections change over the course of the study? How did this differ between the facilities that were newly upgraded and the ones that already had CEmONC capacity? It may be useful to have a figure showing these trends as well.
    - The same goes for blood transfusions: how did these change over the course of the study?
    - “During the intervention period a total of 2,179 CS were performed in the intervention group and 964 in the control group.” (p 11 line 236). This line confuses me because I thought that C-sections were unavailable in the control facilities.

**Response**

- A detail account on the CS was presented elsewhere {*Nyamtema AS, Scott H, Kweyamba E, Bulemela J, Shayo A, Mtey G, Kilume O, and LeBlanc JC, 'Improving Access, Quality and Safety of Caesarean Section Services in Underserved Rural Tanzania: The Impact of Knowledge Translation Strategies', Afr J Reprod Health, 25[3s] (2021), 74-83 DOI: 10.29063/ajrh2021/v25i3s.8]*
- Both interventions and control HCs were already providing CEmONC services. This has been added in the manuscript. Although all HCs provided BT services the study did not capture these data.

14. I appreciate the inclusion of the cost data here, yet given that the methods were not appropriately described in this manuscript and seem to refer wholly to a different manuscript, these are not ‘results’ of this study. They should be moved to the discussion section.
    - The same is true of the “uptake of CEmONC services” section: these are not original findings of this study so should be moved to the discussion section.

**Response**

The authors briefly described the methods used to determine the CEmONC costs on page 8 paragraph 2. … collected from the health centres, Tanzania Medical Store Department and non-governmental organizations that had upgraded health centres for CEmONC services provision. The authors consider the uptake of the project interventions as one of the key results which were set during development of the proposal. In view of that, we had added a few lines in the methods to describe how we implemented our uptake strategies.

Discussion:
- Given the apparent lack of control group for the L&M and BRN measurements, the causal language used in the discussion, i.e. “In this project, improving leadership and management was a change factor, a fuel for progress,” (p 12, line 279) is inappropriate. None of the changes in utilization or quality can be causally attributed to changes in leadership.
- Study limitations need to be acknowledged and discussed.

**Response**

We have edited the sentence and currently reads; “Improvement in these domains could partly be attributed to improved leadership and management”.

**REVIEWER #2:**

1. **General:**
   - Would it be possible to explicitly stat the overall aim and objectives of this study?
   - Was this a stand-alone study or a part of a bigger study (intervention study)?
   **Response**

We stated on page 3 line 70-71(the last paragraph of the introduction) that the objective was to study how to improve access to comprehensive emergency obstetric and newborn care (CEmONC) services in underserved rural Tanzania, where only 12% of HCs were then providing CEmONC. This was a stand-alone study.

2. **Methods**

   Study design: I struggling to understand the study design used in this study. Page4, L86-87: “This study was a prospective cohort study in seven health centres in Morogoro region, Tanzania. Five of these received an intervention and two served as controls in order to detect secular trends”
   - Page 5, L97-100: 20203. “Gairo and Melela HCs (publicly funded) were already providing CEmONC and were included in the intervention group in order to study how CEmONC services could be strengthened. St. Joseph HC represented a group of faith-based organizations. Using simple random sampling of Morogoro HCs, Mlimba and Mkamba HCs were allocated to be control sites.”

   Comments:

   A prospective cohort study is an observation study, participants are either exposed or controls (not exposed). Using the wording such as “…received an intervention”, “…included in the intervention group”, “…were allocated” makes the study an experimental one. Can you please check this out and present the correct study design by specifying (if it’s a cohort) the key features of a cohort design such as exposure status, how long follow up, outcome (s) of interest, etc.
   If it was an intervention study, this should be clearly described and the content of every section should reflect the study design used and clearly specify the key features of an experimental study.

   - On Page 6, there is even a section on “Interventions” consisting of “Capacity building in emergency obstetric and newborn care, and anaesthesia” and “Strengthening leadership and management”
   Comment: Alluding to my comment above, this “Interventions” means the study was not an observational one (cohort), but an experimental (or quasi-experimental) study. Table 1 is also referring to “before and after the intervention in the control and intervention health centres”

**Response**

We have edited the study design. It is a quasi-experimental study

3. Further details on data analysis will also depend on the study design. For instance, did you consider any relative measures of association? How will you know that the “intervention” was effective to show an impact as you mention L176-177 “multiple statistical tests were used to assess the impact of the intervention model”. Data analysis: “multiple statistical tests were used to assess the impact of the intervention model”.
   Comment: Do these “multiple statistical tests refer to One-way ANOVA and Chi-square tests or was there any other test. If there was any other test, I would suggest to describe it. Were there any descriptive statistics done? Would you consider any measures of association as you are assessing the impact of the intervention?

**Response**

We have edited the sentence. The authors used one-way ANOVA and Chi-square tests to assess the impact of the intervention model.

4. **Results:** “Interventions resulted in improved responsibility and accountability among managers”
   Comments: Can you specify the results that substantiate this claim? How did you define “accountability in this manuscript?

**Response**

This sentence has been deleted from the paper. However, the authors assessed the responsibility and accountability among managers using BRN key indicators for the health facility management, use of data for improvement, staff performance assessment, organization of services, handling of emergencies and referral care, health facility social accountability, and infection prevention and control.

5. There are a few typos to be corrected for instance in the referencing style (References 4, 5, 6)

**Response**

Thank you for identifying these typos. They have been corrected accordingly
