# Supplementary material for: Scale up and strengthening of comprehensive emergency obstetric and newborn care in Tanzania
Source: PLoS One. 2022 Jul 8;17(7):e0271282. doi: 10.1371/journal.pone.0271282 (PMC9269945; doi:10.1371/journal.pone.0271282)
Supplement: Attachment — Submitted filename: Response to reviewers 26042022.docx [file pone.0271282.s006.docx]

**RESPONSE TO REVIEWERS’ COMMENTS**

**1. ACADEMIC EDITOR’S COMMENTS**

PONE-D-21-37381R1

Scale up and strengthening of comprehensive emergency obstetric and newborn care  in Tanzania

PLOS ON

6. Review Comments to the Author

Please use the space provided to explain your answers to the questions above. You may also include additional comments for the author, including concerns about dual publication, research ethics, or publication ethics. (Please upload your review as an attachment if it exceeds 20,000 characters)

Reviewer #1: While now described as a 'quasi-experimental' design, the study still does not have a suitable identification strategy to support the claims that are asserted in the results and discussion.

First, the study relies on a too small sample size (5 intervention facilities and 2 control facilities.

**Response**

Intervention HCs were chosen to reflect the diversity of funding models and diversity in accessing care at a referral hospital. The intent was not to have a representative sample of HCs in Morogoro region but, within the funding constraints, choose those centres that would give the greatest understanding of the diverse needs throughout the region. Similarly, the control centres were chosen to reduce the likelihood of contamination from the intervention HCs and to capture the diversity of centres that would not receive an intervention. We purposely chose fewer control HC so that we could allocate more of our funding to thoroughly studying the implementation and scale-up of CEmONC in intervention centres. We primarly followed the control HCs to detect secular trends that could potentially explain some of the differences we saw over time in the intervention HCs.

Second, the results report increases leadership and management in the intervention facilities based on the BRN and the L&M survey, but there is no comparison to the control facilities and no sample size for the L&M survey is provided.

**Response**

It is true that we did not assess L&M skills in control centre staff but this went beyond the scope of the study. We did not have the resources to assess L&M skills in control centres nor the ability to offer them incentives for such intrusive data collection such as offering training after the study was completed. Again, we primarily used control HCs to detect secular trends. This means that this study was primarily a before-after design. The BRN and the L&M survey results were presented as a before and after in the intervention HCs. This is stated in the methods (line 101 – 104) and limitations of the study (line 356 – 362).

Third, where the control facilities are used in the utilization and quality analysis, they find similar if not better improvements than the intervention facilities. The true extent of the differences is difficult to ascertain because there is no formal difference-in-differences analysis and again, the sample size are far too small to warrant statistical comparison. However, none of these secular improvements are mentioned in the discussion: the focus is entirely on the health systems strengthening and scale up. From my read, there is no evidence to support any improvement in the intervention facilities, making these conclusions unwarranted.

**Response**

These are important points and it is difficult to statistically compare HCs that were chosen specifically to capture the diversity of HCs in Morogoro. We have added discussion about the differences in outcomes between intervention and control HCs particularly for fatality rate. Despite increasing complexity of the cases managed at the intervention sites because of lower referral rates, improvements were noted. Other factors were probably at play, as demonstrated by improvements in the control sites as well, but given the increased number and complexity of deliveries managed at the intervention sites, this was felt to be a relevant finding.

**2. Reviewer #3: Please see comments in attachment:**

On page 4:

1) How about the other two HCs in the intervention arm? In line 85, five HCs were referenced and two controls. Please clarify if you had 3 HCs as intervention sites and 2 as control, making 5 all together. It’s a bit confusing to readers.

**Response**

The second sentence on line 85 indicates that the total number of intervention HCs is 5. The same paragraph indicates that of these (five); two (Kibati and Ngerengere) had not started providing CEmONC, and three (Gairo, Melela and St. Joseph) had CEmONC but needed to be strengthened. This description is provided in the paragraph. The authors have added a line that reiterates that there were 5 intervention sites and 2 control sites.

On page 9:
2) How many HCs were involved in the sampling? Please expand a bit more for clarify.

**Responses**

Thank you. The following sentence has been added; *“Mlimba and Mkamba HCs were randomly allocated to the control group from the remaining 5 publicly funded HCs that were already providing CEmONC services.”*

3) Did the authors consider any co-founding variables? If yes, how were these variables controlled for in the analysis?

**Response**

This study had several confounders that could have positively or negatively affected health facility deliveries. These included differences in fertility rates and consequently different population growth rates between the study districts – differences in fertility rates are also likely to make differences in facility delivery; 2) differences in leadership effectiveness at the council level, some may be more assertive than the others; 3) the act of regular sharing of key results during the regional quarterly meetings that included the council with the control HCs could have led to contamination. We could not control these factors. These have been included in the limitations of the study

While revising your submission, please upload your figure files to the Preflight Analysis and Conversion Engine (PACE) digital diagnostic tool, <https://pacev2.apexcovantage.com/>. PACE helps ensure that figures meet PLOS requirements. To use PACE, you must first register as a user. Registration is free. Then, login and navigate to the UPLOAD tab, where you will find detailed instructions on how to use the tool. If you encounter any issues or have any questions when using PACE, please email PLOS at [figures@plos.org](mailto:figures@plos.org).
