# Supplementary material for: Scale up and strengthening of comprehensive emergency obstetric and newborn care in Tanzania
Source: PLoS One. 2022 Jul 8;17(7):e0271282. doi: 10.1371/journal.pone.0271282 (PMC9269945; doi:10.1371/journal.pone.0271282)
Supplement: Attachment — Submitted filename: Responses to reviewers comments.docx [file pone.0271282.s007.docx]

**RESPONSES TO REVIEWER’S COMMENTS**

1. Abstract, Result, sentence: “The case fatality rate decreased slightly from 1.5% (95% CI 0.6–3.1) at baseline to 1.1% (95% CI 0.7-1.6) during the intervention period (not statistically significant).” The term “case fatality rate” may be replaced with “direct obstetric case fatality rate” given the definition provided in the footnote in Table 1 and the content of WHO Monitoring Emergency Obstetric Care – a Handbook (<https://apps.who.int/iris/bitstream/handle/10665/44121/9789241547734_eng.pdf?sequence=1>).

**Response**

Thank you. Since there are direct and indirect obstetric causes of maternal deaths, we have replaced the term with “obstetric case fatality rate”.

2. Abstract: State the aim of the study in the abstract. Is this the aim of the study: To detect secular trends in Health Centres (HC) in Morogoro region of Tanzania following the integration of Accessing Safe Deliveries in Tanzania (ASDIT) project with leadership and managerial capacity building in these healthcare facilities?

**Response**

The aim of the study was to study how to improve access to comprehensive emergency obstetric and newborn care (CEmONC) in underserved rural areas. This is stated in the last sentence of the introduction section of the abstract, and in the last paragraph on page 3.

3. Abstract, conclusion: “Integration of leadership and managerial capacity building, with CEmONC-specific interventions has resulted in health systems strengthening and improved quality of services.” Consider revising the statement to read: “Integration of leadership and managerial capacity building with CEmONC-specific interventions was associated with health systems strengthening and improved quality of services.” This means replacing the words “has resulted in” with “was associated with.”

**Response**

We have replaced the words “has resulted in” with “was associated with.”

4. In material and methods, first paragraph, after the sentence “Five of these received an intervention and two served as controls in order to detect secular trends” include the following: The HCs in the intervention group were Kibati, Ngerengere, Gairo, Melela and St. Joseph HCs.

**Response**

Thank you. This sentence has been added in the manuscript.

5. Materials and methods, “The theory of change: a model formulation”: (a) “In order to develop a set of sound and scientifically derived interventions the project applied principles of operations research to identify and address operational factors that determine maternal and newborn health care in Tanzania.” Provide a reference for principles of operational research. (b) “Using evidence-based science on the interventions that work, …” Provide a reference for evidence-based science on the interventions that work.

**Response**

Four references have been inserted.

6. Materials and methods, Strengthening leadership and management, sentence: “The workshops were conducted in 2018 and 2021 and involved participants from 20 primary health facilities, i.e., the 5 intervention health centres and 15 satellite dispensaries,...” Explain the referral relationship between the 5 intervention health centres and 15 satellite dispensaries.

**Response**

The following sentence has been added; *“These dispensaries referred patients with medical complications to the study health centres*.”

7. Materials and methods, Data collection, sentence: “The BRN tool assesses the following domains: 1) health facility management (12 indicators)…” The indicators are difficult to find in references 17 and 19 cited by the authors. Are you referring to Model of Care Initiative in Nova Scotia (MOCINS) Process Indicators or MOCINS Outcome Indicators contained in reference number 19? To avoid confusion, present the indicators in a table.

**Response**

The domains and indicators are for the Tanzanian Big Results Now assessment tool. These indicators do not refer to Model of Care Initiative in Nova Scotia (MOCINS) Process Indicators or MOCINS Outcome Indicators contained in reference number 19. Since the table with domains and indicators is too long, (Table 1. Big Results Now Star Rating domains and indicators), we have provided it as an additional information to this manuscript … however, the legend has been inserted in the manuscript.

8. Materials and methods, Data collection, sentence: “The L&M survey primarily used Likert scales to assess data on care providers’ perceptions on L&M competencies,…” The questionnaire (the questions and the scales) that was used for the assessment should be described in a table.

**Response**

A table has been inserted. Table 2. Leadership and managerial domains assessed in 2018 and 2021

9. One-way ANOVA and Chi-square tests were used. No p-value was stated in the results. Explain.

**Response**

Thank you for observation. The confidence interval set to 95% CI for both parametric and non-parametric variables. We have replaced the words “p-value at < 0.05” with “95% CI” in the Methods: data analysis section.

10. The term “case fatality rate” may be replaced with “direct obstetric case fatality rate” and defined in the materials and methods section. This will involve replacing “case fatality rate” with “direct obstetric case fatality rate” in the footnote in Table 1. This revision will be in line with the terminology changes in the WHO Monitoring Emergency Obstetric Care – a Handbook (<https://apps.who.int/iris/bitstream/handle/10665/44121/9789241547734_eng.pdf?sequence=1>).

**Response**

As reported above (comment no. 1), we have revised all places where the term ‘case fatality rate’ appeared.

11. Results, Strengthening health systems, sentences: “Capacity-building strategies in transformational leadership and change management resulted in improved leadership and management as assessed using the BRN star rating assessment system and the survey. Capacity building contributed to improved health facility performance and maternal and child health outcomes.” These are interpretation of the results. Therefore DELETE the sentences.

Response

The two sentences have been deleted

12. In Figure 2, there are black and orange coloured horizontal lines. Are these confidence intervals. Specify.

**Response**

Thank you. We have added in the key (footnote) below the figure that “black and orange coloured horizontal lines are 95% CI”.

13. Results, Strengthening health systems, sentence: “The sub-scales included vision, support, task orientation and role clarity.” Explain how these indicators were improved. Include the accompanying data.

Response:

Thank you. Instead of presenting data on the team climate subscales we have decided to present data on the L&M overall domains that were assessed in this study, i.e., 1) team climate of facilities; 2) staff role clarity; and 3) job satisfaction. These domains are also presented in table 2. Leadership and managerial domains assessed in 2018 and 2021.

14. Results, Strengthening health systems, sentence: “In 2021, the overall BRN ratings increased in 15 (79%) of the nineteen primary health care facilities,…” This is difficult to understand because in the materials and methods 20 (and not 19) primary health care facilities were mentioned.

**Response**

Although capacity building in L&M and all other assessments involved 20 facilities, the 2018 BRN star rating assessment was not done in one HC. We have added a sentence to clarify it in the results – section.

15. In Figure 3, what does the dotted line represent? Is it the overall trend in the referral rate in the intervention HCs?

**Response**

The dotted line represented the linear trend in the referral rates in the intervention HCs. However, to avoid confusion, we have removed it.

16. Results, Utilization of CEmONC services, sentences: “For instance, the mean monthly deliveries at Gairo and St. Joseph HCs increased from 71 (67 – 76) to 137 (124 - 150) during intervention period, and from 48 (41 – 55) to 129 (116 - 143) respectively. The mean monthly deliveries at Kibati and Ngerengere increased from 21 (18 – 23) to 34 (30 – 37) during intervention period, and from 26 (23 - 28) to 33 (31 – 36) respectively.” Specify the meaning of the numbers in bracket.

**Response**

The numbers in the brackets signify 95% CI. These have been added in the manuscript.

17. Results, Quality of CEmONC services: What were the primary causes of the maternal deaths and the avoidable/modifiable factors associated with them (at least in the intervention HCs).

**Response**

We have added the following sentences in the manuscript. “During these periods (baseline and during the intervention) 40 maternal deaths occurred. The primary causes of these deaths during the intervention period were postpartum haemorrhage 8, pre/ eclampsia 5, puerperal sepsis 3, complications of anaesthesia 2, uterine rupture 2, severe anaemia in pregnancy 2 and antepartum haemorrhage 1. The causes of deaths were not established in 13 maternal deaths that occurred at baseline, and four that occurred during the intervention in the control HCs because of inadequate records keeping. Avoidable factors were determined in only 87% i.e., 20 cases out of 23 deaths because the case files for the other 3 had inadequate information. Delay to provide appropriate care after reaching the facilities was identified in 80% i.e., 16 of 20 facilities. A delay in seeking treatment/ reaching the facility was found in 55% i.e., 11 out of 20 deaths.”

18. Results, The requirements and costs of scaling up of CEmONC services in health centres, sentence: “Detailed findings on the requirements and costs for scaling up CEmONC in health centres in Tanzania are reported elsewhere.” Reference 19 was cited by the authors. However, the word Tanzania could not be found in reference 19 (i.e. Model of Care Initiative in Nova Scotia (MOCINS): Final Evaluation Report).

**Response**

We have replaced the reference with the appropriate one.

19. Discussion, Health systems strengthening for maternal and newborn health care, sentence: “Strengthening leadership and management at the health facility district and regional health system levels resulted in strengthened health systems building blocks, which are vital for provision of effective services.” It is preferrable to use the words “was associated with” rather than “resulted in.”

**Response**

Replacement done. Thank you

20. Discussion, Scale up of CEmONC services, first paragraph, sentence: Improving the availability and access to comprehensive emergency obstetric and neonatal care services resulted in a marked increase in utilization of services (including women with obstetric complications) and reduced referral rates to distant hospitals in intervention centres. It is preferrable to use the words “was associated with” rather than “resulted in.” This is because the improvements could have been due to other factors such as changes in human migration and population. These may explain some of the outcomes in the control HCs.

**Response**

Replacement done. Thank you

21. Discussion, Scale up of CEmONC services, second paragraph, sentence: “Maternal mortality also dropped in the two control centres but they continued to refer pregnancies at the same rate to secondary hospitals, suggesting little change in the complexity of pregnancies and deliveries they were managing.” The data on complexity of the cases managed at the intervention and control HCs were not presented in the result section.

**Response**

Thank you. By saying the complexity of pregnancies and deliveries the authors meant complicated pregnancies managed at the health facilities. In the results in table 3, the authors presented a summary of women who had obstetric complications (maternal morbidities) at baseline and during the intervention period. In the Results, Utilization of CEmONC services (sub-section): we also presented figures of women with obstetric complications referred to the nearby district hospital. We thought that the data on maternal morbidities and referred pregnant women provided a snapshot of complexity of pregnancies and that somehow justify our statement.

22. Discussion, Scale up of CEmONC services, fourth paragraph, sentence: “Findings from previous and current studies coupled with effective knowledge translation strategies, engagement, political will and commitment resulted in a nation-wide scale up of CEmONC services in public health centres.” Delete “and current.”

**Response**

The words are deleted

23. Discussion, “Addressing the fear of the unknown: the cost of scaling up CEmONC services,” sentence: “The requirements and related costs reported in this study fill the existing vacuum of science and knowledge.” Delete this sentence.
    This is because the authors also wrote in the results section that “Detailed findings on the requirements and costs for scaling up CEmONC in health centres in Tanzania are reported elsewhere.” Therefore the index report/study can’t be filling any vacuum in knowledge. Additionally, there are previous studies on the cost of scaling up a health facility in low- and middle-income countries.

**Response**

The sentence is deleted

24. Discussion, Limitations of the study: Acknowledge that other factors such as changes in human migration and population could have affected the findings in both intervention and control HCs.

**Response**

Thank you. These factors have been added in the list of limitations

25. Conclusion: “Integration of leadership and managerial capacity building, with CEmONC-specific interventions has resulted in health systems strengthening and improved quality of services.” It is preferrable to use the words “was associated with” rather than “resulted in.”

**Response**

The words are replaced. Thank you
